# Intravascular Lymphoma in the CNS: Options for Treatment

**DOI:** 10.1007/s11940-017-0471-4

**Published:** 2017-08-23

**Authors:** Damir Nizamutdinov, Nitesh P. Patel, Jason H. Huang, Ekokobe Fonkem

**Affiliations:** 1 College of Medicine, Texas A&M Health Science Center, Temple, TX USA; 2 Department of Neurosurgery, Baylor Scott & White Health, Temple, TX USA

**Keywords:** Intravascular lymphoma, CNS, Treatment

## Abstract

*Purpose of review* The purpose of this review was to discuss therapeutic manipulations and effective current interventions available to treat intravascular lymphoma in the central nervous system.

*Recent findings* Patients experienced resolution and remission of disease for 14 months and up to 2 years after eight cycles of R-CHOP and four courses of intrathecal therapy with MTX, cytarabine, and prednisolone. Intravascular use of unfractionated heparin during therapy may contribute to better outcome.

*Summary* Series of therapeutic avenues were analyzed and compared. The effective current treatment of intravascular lymphoma in the CNS is considered to be a combinational intrathecal methotrexate-based chemotherapy with rituximab. Since intrathecal administration bypasses the blood–brain barrier, lower doses can be given, which thereby minimizes systemic toxicity. Practical use of intrathecal chemotherapy is also justified for prophylaxis in intravascular lymphoma-diagnosed patients without CNS involvement.

## Introduction

Intravascular lymphoma (IVL) is a rare form of extranodal non-Hodgkin’s lymphoma that selectively involves the lumina of small blood vessels, especially capillaries [1]. The disorder was initially characterized in 1959 as a neoplasm of vascular endothelial cells and was thereby named “angioendotheliomatosis proliferans systemisata” [2]. However, the lymphoid origin was elucidated by immunohistochemistry in 1986; at that time, the disorder was renamed “angiotropic large cell lymphoma,” and the nomenclature was further refined to “intravascular lymphoma” by the World Health Organization in 2008 [3, 4]. While the majority of IVL is of the B lymphocyte lineage, rare cases have been observed involving the natural killer (NK)/T cell lineage [5].

Given the intravascular (IV) proliferation of neoplastic lymphocytes, the disorder typically presents with signs and symptoms of vascular occlusion and thus can have a variable clinical presentation depending on the specific site of the body that is affected [6••]. In 2014, Fonkem et al. performed a meta-analysis of 740 IVL cases and showed that the central nervous system (CNS) is the most common location (41%) of primary disease [7••]. However, previous reports suggested that clinical manifestations also vary based on a patient’s geographic origin. Specifically, involvement of the CNS and skin is observed at a greater frequency in Western countries, whereas patients in Asian countries typically present with fever, hematophagocytic syndrome, hepatosplenomegaly, and bone marrow invasion [8].

All cases of IVL are managed as disseminated disease due to the IV nature of the disorder, and the most common treatment regimen consists of cyclophosphamide, doxorubicin, vincristine, prednisone, and rituximab (R-CHOP) [1]. Clinical evidence for this combination is mainly derived from case reports and retrospective studies, and long-term survival evidence has been limited due to the short follow-up in many of these studies [1, 9–13]. Furthermore, given that systemic treatment with R-CHOP does not adequately penetrate the CNS, further CNS-directed intervention is warranted given that approximately 30–40% of patients have CNS involvement at the time of diagnosis and another 25% develop CNS involvement during the follow-up period [14]. This review mainly focuses on therapeutic options and managements of IVL in the CNS.

## Methods

Literature search: We conducted a literature search of PubMed from 1962 to March 2017 using the following terms: intravascular with lymphoma or lymphomatosis, angioendotheliomatosis, endotheliomatosis, and large cell lymphoma with angiotropic or angiotrophic. Case reports of IVL with CNS involvement were included, but reports that did not comment on therapy of CNS involvement or patient outcomes were excluded. Publications in a non-English language were also excluded from this review.

## Clinical manifestations and diagnosis

### Clinical manifestations

Clinical presentation of IVL with CNS involvement depends on location and severity of cerebrovascular pathology associated with disease. Aforementioned pathology compromises optimal blood supply to important brain structures and results in impaired brain function. IVL limited only to the CNS is an extremely rare condition. In 2016, Fonkem et al. performed a meta-analysis of 654 IVL cases with neurological presentations and reported that 52% of all IVL-diagnosed patients were presented with neurologic symptoms and neurologic complications [15]. Of them, 82% were presented with CNS symptoms, 18% with peripheral nervous system (PNS) deficits. Most commonly observed CNS complication was cognitive impairment (60.9%), including encephalopathy and dementia. Paralysis (upper motor neuron) and paraplegia were second most commonly reported CNS symptoms (22.1%). Seizures, including myoclonic jerks and status epilepticus, were third most common clinical symptoms (13.4%) of CNS involvement [15]. Among other reported symptoms were vision disturbances (8.7%), ataxias (7.6%), stroke-like symptoms (7.6%), headaches (6.9%), myelopathies (5.8%), dysarthrias (5.4%), sensory and hearing deficits (4.7 and 4.4%, respectively), and others [15].

### Diagnosis

Unfortunately, due to lack of diagnostic algorithms and very diverse clinical presentation, 60% of IVL patients with CNS involvement are diagnosed postmortem [15, 16]. IVL is rare disease, but when a patient demonstrates rapid development of progressive neurological manifestations without logical correlation to previous history of disease, IVL should be suspected until more clinical diagnostic tests are performed.

Radio-diagnostic criteria found useful in diagnostics of IVL with CNS involvement. Neuro-radiological images are characterized by absence of uniform radiological findings. Thus, MRI should be routinely made to all suspects to rule out more common neurologic disease. Neurosurgical biopsy performed in timely manner is highly recommended and serves as an accurate diagnostic tool, but should be used sparingly in the brain, due to invasiveness [15, 17]. Since IVL is systemic pathology, biopsy from other easily accessible sites should be considered [17]. Laboratory findings reveal a pathologic hemogram in most patients. Usually, thrombocytopenia (29%) or leukopenia (24%) does not occur without developed anemia (63%) [17]. Increased serum lactic dehydrogenase and beta-2 microglobulin levels were observed in more than 80% of patients [9]. Combination of diagnostic approaches is needed to timely confirm a diagnosis and start adequate therapeutic regimen.

## Therapeutic interventions

### General approaches

Due to the rarity of IVL, no prospective, randomized trials have been performed to evaluate the optimal treatment of CNS involvement; instead, support for current methods is derived from case reports, retrospective studies, and trials of more common lymphomas affecting the CNS [18].

Intrathecal chemotherapy involves injection of chemotherapeutic agents directly to cerebrospinal fluid within the intrathecal space, thereby eliminating the need for the drugs to cross the blood–brain barrier (BBB). This method has been employed in recent cases of IVL as both CNS treatment and prophylaxis [19–21•, 26]. The combination of methotrexate (MTX), cytarabine, and prednisolone given intrathecally has been shown to be effective in various degrees of CNS involvement [20, 21•, 23]. In 2014, a Japanese patient with pontine involvement was the first reported case of IVL involving the CNS that achieved remission with only R-CHOP and intrathecal therapy. The patient experienced resolution of the pontine lesion and achieved remission for 14 months after eight cycles of R-CHOP and four courses of intrathecal therapy with MTX, cytarabine, and prednisolone [20]. More recently in 2016, a patient that developed IVL with involvement of the dura on brain magnetic resonance imaging (MRI) achieved complete remission for 2 years without CNS relapse after a similar treatment regimen [23]. Furthermore, a patient with IVL involving the cauda equina causing lower extremity paraplegia achieved complete remission for 28 months with the same R-CHOP and intrathecal chemotherapy combination, but her paraplegia persisted [21]. Intrathecal administration appears to be a good option for cauda equina involvement given that two other patients presenting with cauda equina syndrome both achieved clinical improvement and complete remission following intrathecal chemotherapy and systemic treatment with either R-CHOP or R-CPADEM (rituximab, MTX, doxorubicin, endoxan, lederfolin, and prednisone) [19, 22].

Prophylaxis with intrathecal chemotherapy has resulted in varied outcomes [24–27]. Two cases of IVL without CNS involvement at diagnosis achieved complete remission without CNS relapse for 20 months in the patient that received four doses of intrathecal MTX and 21 months in the patient that received intrathecal MTX, cytarabine, and dexamethasone [25, 26]. However, even though a patient with IVL of the penis, prostate, and bones without CNS involvement at diagnosis achieved complete remission after six cycles of systemic treatment with R-CHOP and four doses of intrathecal MTX for CNS prophylaxis, the patient ultimately experienced CNS relapse after 6 months [24]. Overall, intrathecal chemotherapy has been effective as both treatment and prophylaxis of CNS involvement of IVL. Benefits of the intrathecal method include smaller drug doses due to the small volume of distribution of the CSF, which thereby minimizes systemic toxicity. Given that this treatment combination is better tolerated than other regimens, it may be an appropriate choice for treatment of elderly patients or patients in poor medical condition that cannot tolerate regimens with greater toxicity.

Despite the benefits of intrathecal chemotherapy, some patients with CNS involvement have responded to R-CHOP treatment alone [27–29]. A patient with IVL of pituitary gland who presented with features of pituitary apoplexy and ophthalmic symptoms achieved clinical and radiographic improvement with three cycles of R-CHOP. Although the patient continued to have right cranial nerve III palsy, the sellar mass resolved after R-CHOP therapy, indicating that IVL of the pituitary can be treated with R-CHOP alone [27]. In 2014, a Japanese patient without neurological symptoms was diagnosed with IVL of the pons after at T2 weighted brain MRI showed a pontine hyper-intensity. After eight cycles of R-CHOP without intrathecal therapy, the patient experienced resolution of the pontine lesion without recurrence for 5 years [28•]. This case indicates that R-CHOP is effective at treating IVL with early CNS involvement. Furthermore, it should be noted the patient also had taken verapamil for paroxysmal supraventricular tachycardia. Verapamil inhibits p-glycoprotein, which is an important mediator of resistance to anticancer drugs and also limits drug penetration through the BBB [30, 31]. Thus, early initiation of chemotherapy in addition to a p-glycoprotein inhibitor like verapamil may be important in treating IV large B cell lymphoma with CNS involvement and is an area of possible study [28•]. However, another patient with pontine involvement of IVL experienced diminution of her pontine lesion after three cycles of R-CHOP alone and this patient was not taking verapamil concurrently [29]. While the utility of verapamil is questionable, these cases suggest that IVL of the pituitary and pons responds well to R-CHOP alone. Despite this, a case of IVL involving both the brain and spinal cord initially responded to five cycles of R-CHOP, but after 5 months, the patient experienced CNS recurrence with neurological symptoms, suggesting that R-CHOP alone may not be sufficient treatment for IVL with extensive CNS involvement [32].

While R-CHOP seems beneficial in cases of less extensive CNS involvement, especially of the pituitary gland and pons, CHOP therapy alone without rituximab has mostly led to poor outcomes [33, 34]. Two patients presenting with memory loss and behavioral changes with involvement of the brain on MRI both expired after treatment solely with CHOP, suggesting the need for CNS-directed therapy [33, 34]. One of the patients initially received high-dose IV methylprednisolone for 5 days, which temporarily improved her neurological condition from a Glasgow coma scale (GCS) score of 7 to GCS of 11 [34]. Accordingly, most other cases utilizing high-dose corticosteroids have only observed a transient improvement in neurological condition and thus should not be the sole component of CNS treatment [18, 35, 36].

In 2012, a case of IVL, affecting the pons and left temporomesial area in a Caucasian male, was treated with the Bonn protocol, which consists of six cycles of high-dose MTX, ifosfamide, procarbazin, cytarabine, vinca alkaloids, and dexamethasone in addition to rituximab [37, 38]. The patient achieved complete clinical and radiographic remission for 29 months since diagnosis after receiving treatment, and although the protocol was developed for primary CNS lymphoma (PCNSL), the favorable outcome suggests that a high-dose MTX-based polychemotherapy is appropriate for treatment of IVL in the CNS [37]. Another regimen that has shown promise was recently reported by Prat-Martin et al. and consists of carmustine, cytarabine, and MTX. In this report, a case of IVL affecting the brain achieved complete remission for 17 months since diagnosis after two cycles of carmustine, cytarabine, and MTX, followed by one cycle of R-CHOP and stem cell support [39]. The favorable CNS response may be as result of the high penetration ability of BBB by carmustine, cytarabine, and MTX.

### Radiotherapeutic approaches

PCNSL is often treated with high-dose MTX followed by whole-brain radiotherapy, and although a few case reports have suggested treating the CNS involvement of IVL in a similar manner, the outcomes have been conflicting [35, 40]. In 2012, a patient with IVL limited to the CNS experienced radiographic and clinical improvement after three cycles of high-dose MTX followed by whole-brain radiotherapy at a total dose of 30 Gy; however, the patient expired 6 months after disease onset [40]. Conversely, a patient with IVL of the dura on brain MRI was successfully treated with involved field radiotherapy after resistance to high-dose MTX [35]. While radiotherapy alone is not commonly employed in the therapy of CNS IVL, a patient with recurrent stroke-like symptoms experienced stabilization of intracranial lesions for 7 months after therapy with R-CHOP, MTX, and cranial radiotherapy [41]. However, a patient that received a total of 45 Gy via whole-brain radiotherapy followed by R-COP (rituximab, cyclophosphamide, vincristine, and prednisone) experienced neurological deterioration 10 months after onset despite clinical and radiological improvement for 8 months since onset. The lack of CNS-directed chemotherapy in combination with radiotherapy in this patient could explain the clinical course, suggesting that intrathecal chemotherapy has greater utility than radiotherapy [42].

### Novel therapeutic avenues

Recent case reports have presented novel treatment options for the CNS component of IVL [24, 43]. In the aforementioned case of failed CNS prophylaxis with intrathecal MTX in the patient with IVL of the penis, prostate, and bones, CNS relapse significantly improved after three cycles of oral temozolomide with IV rituximab and high-dose MTX [24]. Also, in 2015, Yoshida et al. used IV unfractionated heparin and observed rapid improvement of the patient’s white matter and auditory nerve lesions with improved hearing after 10 days of heparin initiation; furthermore, the patient achieved complete remission after five cycles of R-CHOP and intrathecal MTX [44••]. Prior studies have shown that IVL may express heparin-responsible adhesion molecules such as L-selectin and very late antigen-4; thus, treating with heparin could prevent the aggregation of the neoplastic lymphocytes within vascular lumina and thereby contribute to the treatment of CNS involvement without further contributing to the systemic toxicity of chemotherapeutic agents [44••, 45].

## Conclusion

At the present time, the most effective treatment of IVL in the CNS is a combination of MTX-based intrathecal chemotherapy and R-CHOP [19–26]. The lower dose of administered drug can be used due to BBB penetration, and resulted in minimized systemic toxicity. Intrathecal chemotherapy should also be utilized as prophylaxis in patients without CNS involvement.
